# Impact of a community-based participatory research project with underserved communities at risk for hepatitis C virus in Ho Chi Minh City, Vietnam: an evaluation study

**DOI:** 10.1186/s40900-024-00619-6

**Published:** 2024-08-07

**Authors:** My Nguyen Le Thao, Giang Nguyen Quoc, My Do Thi An, Hieu Nguyen Minh, Son Pham Hong, Anh Hoang Thai, Phung Than Thi, Vy Nguyen Thuy Thanh, Ngoc Tran Thi, Thuan Nguyen Minh, Barnaby Flower, Graham S. Cooke, Mary Chambers, Jennifer Ilo Van Nuil

**Affiliations:** 1 Oxford University Clinical Research Unit, Ho Chi Minh City, Vietnam; 2 CBPR Community Advisory Groups, Ho Chi Minh City, Vietnam; 3 Department of Infectious Diseases, Imperial College London, London, UK; 4 Centre for Topical Medicine and Global Health, Nuffield Department of Medicine, University of Oxford, Oxford, UK

**Keywords:** Participatory research, community based, Community-based participatory research, Hepatitis C, Health care seeking behavior, Vietnam

## Abstract

**Background:**

Participatory approaches have become a widely applied research approach. Despite their popularity, there are many challenges associated with the evaluation of participatory projects. Here we describe an evaluation of a community-based participatory research study of underserved communities in Ho Chi Minh City (HCMC), Vietnam at risk for hepatitis C virus. The goals of our evaluation were to explore the main benefits and challenges of implementing and participating in a participatory study and to describe study impacts.

**Methods:**

We conducted two meetings with leaders and members of the participating groups followed by in-depth interviews with 10 participants. We then held a dissemination meeting with over 70 participants, including the representatives of each group, researchers from non-governmental organizations (community-based, national and international), and govenrment officials from the Vietnam Ministry of Health and the Department of Health of HCMC.

**Results:**

Results include four categories where we describe first the participatory impacts, followed by the collaborative impacts. Then we describe the benefits and challenges of creating and belonging to one of the groups, from members’ and leaders’ points of view. Finally, we describe the key suggestions that participants provided for future research.

**Conclusion:**

In conclusion, the evaluation approach led to both a research reflection on the ‘success’ of the project and enabled participants themselves to reflect on the outcomes and benefits of the study from their point of view.

**Supplementary Information:**

The online version contains supplementary material available at 10.1186/s40900-024-00619-6.

## Abstract (plain language summary)

Participatory approaches in research aim to include participants in an array of aspects of the study, including developing research questions, collecting data, conducting analysis, etc. It has become a more popular method, however there are still challenges surrounding the evaluation of these projects. Here we describe an evaluation of a community-based participatory research study of underserved communities in Ho Chi Minh City (HCMC), Vietnam at risk for hepatitis C virus. The goals of the evaluation were to discuss and explore the main benefits and challenges with those who participated, as well as assess study impacts. To conduct the evaluation, we conducted two meetings with leaders and members of the participating groups followed by interviews with 10 people who were involved. The evaluation results included four categories including impacts for members as well as wider impacts in the community. Then we describe the benefits and challenges of creating and belonging to one of the groups, from members’ and leaders’ points of view. Finally, we describe the key suggestions that participants provided for future research. In conclusion, the evaluation approach led to both a research reflection on the ‘success’ of the project and enabled participants themselves to reflect on the outcomes and benefits of the study from their point of view.

**Supplementary Information:**

The online version contains supplementary material available at 10.1186/s40900-024-00619-6.

## Background

Participatory approaches have become a widely applied approach. The history of these approaches stretches back to grassroots and liberation movements and crosses disciplinary boundaries of many social sciences [1–3]. The unifying aims of these approaches include addressing social injustice, power struggles, oppression, liberation, marginalization and to support a variety of local activist causes by incorporating partnerships, participation, and action [1]. To date, there are many forms of these approaches, for example, critical participatory action research (CPAR) to address forms of social injustice [1], community-based participatory research (CBPR) to identify and solve health-related issues [4], participatory rural appraisal to improve basic life conditions in communities [5], to name a few. The approaches have been utilized by various sectors including health, education, development, among others [2].

Despite its popularity as an approach, there are many challenges associated in the evaluation of participatory projects leading researchers to many questions on the following topics: defining success in participatory projects, including accounting for different definitions of success, and determining whose version of success “counts,” as well as assessing if results are “good enough” [2, 6–8]. Beyond these broad points about evaluation, there are also documented challenges associated with how to evaluate the specific relationships formed as part of the process [7] and how to assess if partnerships were indeed successful [6]. The meaning and levels of participation as defined by various participants can also be a challenge to describe [2]. The participation goals of these projects are often termed as “deep” engagement but it remains a challenge to assess what this means in specific projects and for different actors in the process.

With calls for research to provide (concrete) impact indicators of the research with communities and/or policies more generally, challenges often arise due to project timing compared with actual observable impact, how to attribute such impact to specific research and not to other factors, and issues related to each context [9]. The idea of impact, or co-impact [10, 11] or co-production of impact [11], and demonstrating it, becomes more difficult when using participatory action research (PAR)/CBPR approaches where the cyclical nature of the approaches (as opposed to linear approaches in “traditional” research) make the impact less distinct [11]. Scholars using these approaches have offered some alternatives, for example, separating findings-based impact and process-based impact [10]. Further, Banks (2017) describes three forms of impact that could occur in participatory projects, including (1) Participatory impacts (usually process-based impacts) described as changes in individuals/institutions who are part of the project; (2) Collaborative impacts, which are based on the findings of the research and used to make changes to practice/policy as well as potentially influencing attitudes and cultures. These impacts tend to be more findings-based (as opposed to process-based); (3) Collective impacts, which are higher level strategies to target changes in practice and policy, which are based directly on the research findings [11].

Based on the characteristics of CBPR approaches and considering these definitions, we designed and conducted an evaluation of a study that used a CBPR approach. The aims of the evaluation were to explore the main benefits and challenges in implementing and participating in a participatory study with underserved communities in Ho Chi Minh City (HCMC), Vietnam and to identify and describe various forms of impact.

In the CBPR study, we focused on exploring access to viral hepatitis care and treatment, with particular attention to hepatitis C virus (HCV). Globally, viral hepatitis accounted for approximately 1.1 million deaths in 2019 of which 96% were attributed to hepatitis B virus and HCV. There continues to be 1.5 million new chronic HBV infections and 1.5 million new chronic HCV infections per year [12]. Despite the burden of viral hepatitis, the World Health Organization (WHO) has ambitious goals to eliminate it as a major public health threat by 2030 [12]. While hepatitis B virus can be prevented through vaccination, HCV prevention relies on public health measures, although cure can be obtained through treatment. The treatment efficacy for HCV has improved dramatically with the introduction of direct acting antivirals in 2015 and access to treatment has increased 10-fold since its introduction with over 9.4 million people accessing treatment for HCV cure between the period of 2015–2019 [12]. Even with access to direct acting antivirals improving globally, there are still missing populations that have yet to be diagnosed, treated and cured [13]. Vietnam provides a good example: a recent meta-analysis estimated HCV prevalence in the general population at only 0.26% but massively higher in specific populations, for example, 16.8% in dialysis patients and up to 57.8% in people who inject drugs (PWID) [14]. Until these populations have been identified, linked to care and treatment, and ultimately cured of HCV, the 2030 elimination goals will not be feasible. Alternative strategies are needed to engage these populations in care and treatment.

In collaboration with Imperial College London, the Oxford University Clinical Research Unit (OUCRU) in Ho Chi Minh City and Hanoi, the Hospital for Tropical Diseases in HCMC, the National Hospital for Tropical Diseases in Hanoi, we have now conducted a cohort study and two HCV treatment trials in HCMC and Hanoi [15, 16]. Among the early trial populations, some high-risk groups for HCV were underrepresented, including PWID. We, as OUCRU researchers, wondered why these groups were not presenting at the hospital, who were the other potential groups not engaged in care, and how could we begin to understand this gap.

We developed a protocol using a CBPR approach to collaborate with communities to identity issues and develop feasible community-led solutions based on the approach described by Israel and colleagues [4]. We wanted to explore themes surrounding access to care for underserved groups at risk for HCV in HCMC, Vietnam, as well as defining who exactly was “underserved” as starting points to understand the barriers and facilitators to care and treatment. Ultimately, we aimed to work with these groups in order to make changes that would improve their access to care [17]. The set-up of the project [18] and the full descriptive results (forthcoming) are described elsewhere, however, we present some details of the main project here to contextualize the evaluation results presented. First, to identify the underserved populations at increased risk for HCV, we consulted with members of two stakeholder groups that were formed in the early phases of the project: the Community Advisory Group (CAG) and the Stakeholder Working Group (SWG). Together we identified three groups including men who have sex with men and transgender individuals (MSMTG), PWID, and people who have limited financial resources (LFR). We also identified community activators (CAs) from the CAG who would lead the individual groups. The individual members of the three groups were approached and invited to join the study by the CAs after a series of stakeholder meetings starting in September 2020. The partnerships in this study included the two advisory groups, the three CBPR groups, and the OUCRU academic researchers. The three groups were formed in early 2021 and each group went through several cycles of CBPR until June 15, 2022.

## Methods

We used a descriptive qualitative approach, including a variety of methods, in the evaluation component. This design was appropriate because we wanted to explore the meaning and experiences people had during their time with the specific CBPR groups [6]. Additionally, in the main CBPR study, we worked with three groups of 10 to 12 members each so quantitative measures would not provide enough data for more detailed analyses [19]. Throughout the entire study, two OUCRU project research assistants collected handwritten fieldnotes from the groups meetings and activities that they attended to obtain a general sense of the project over time. Then, after the groups finished the CBPR cycles, we integrated three formal evaluation components: (1) evaluation meetings, (2) in-depth interviews, and (3) reflection time at the project dissemination meeting. Each are described below.

We conducted the evaluation component separately from the main study and it was led, with input from the CAs, primarily by the OUCRU team, unlike the main project, which was community-led, with support from the OUCRU team. The evaluation component was conducted by two experienced OUCRU research assistants who had been part of the project since the start. Both have training in qualitative data collection and analysis, as well as in facilitating meetings and discussions. The engagement of researcher partners is fully described in the Guidance for Reporting Involvement of Patients and the Public, short form (Additional File 1).

### Evaluation meetings

The evaluation meetings and reflection activities were co-designed with the CAs and the OUCRU research team. We held two half-day evaluation meetings, one with CAs and another with members from the three groups. We invited all CAs and members from the groups to join. For the first activity of the CA evaluation meeting, the CAs worked in small groups to define and discuss what they thought the results of the study were and then reported back to the full group. The open-endedness of the activity allowed us to determine how each CA percieved and prioritized the outcomes in relation to their main goals. For the second activity, the CAs documented keywords regarding their experiences while implementing CBPR with their groups, which would serve as starting points for sharing what they found most interesting, helpful and/or challenging about participating as a leader. The third activity focused on the level of participation and engagement that the CAs thought they had contributed personally. For the fourth activity, the CAs were encouraged to reveal their own journeys with the project by sketching a ‘river of life’ [20] to show the main activities and highlight their personal achievements. Finally, in the fifth activity, the CAs illustrated, in a drawing, what they thought the main influences and impacts this study had on their communities.

For the second evaluation meeting with CBPR members, we started by showing the data from the CA session and asked them to add or comment. The next activity focused on members’ experiences in the CBPR project by asking them to write keywords about their experiences and feelings when applying CBPR to conduct research within their groups. The following actitivites explored (1) their level of engagement to each category of activities and (2) the ‘river of life’ exercise (both mentioned above). In the last activity, members were encouraged to sketch their expectations about the future, starting from drawing a simple object that they believed linked and enabled them to express their dreams and hopes related to the project.

### In-depth interviews

We collected individual in-depth interviews with advisory group members, CAs, and CBPR members. We used convenience sampling to recruit for the interviews and aimed to interview 9–12 participants. We stopped recruiting after the interviewers felt they had collected a range of experiences. We used a semi-structured interview guide that focused on participants’ experiences of the CBPR practices within their groups and their perceptions about the project over the two-year research period. We also developed questions aimed at collecting their thoughts regarding implementing research in their wider communities, as for most of them, this was their first time. Finally, we explored the impacts they thought this research had on their communities and their suggestions for future collaborations. These topics formed the questions on the interview guide and we used probing when required to explore the topics in more detail. After each interview, we adjusted the guide as needed for subsequent interviews.

### Reflection at dissemination meeting

Finally, we included time for reflection at the study dissemination meeting, that included policy makers, government stakeholders and group members. The research assistants took fieldnotes on these reflections. In this meeting, the CBPR members presented their groups’ research findings and we had disscussions among all participants on related issues. We also held a photo exhibit of images taken by the members of the CBPR groups related to their care seeking and life experiences more broadly. The CBPR groups also created a short film about HCV care seeking that they screened during the meeting.

### Analysis

Data for this analysis included fieldnotes from the duration of the study, meeting notes from the evaluation meetings, the river of life drawings, and interview transcripts. We read the data multiple times, first to familiarize ourselves with the full dataset and to highlight the main points. Then we conducted more detailed coding on the full transcripts using an open coding process and creating a codebook. Finally, we categorized the coded data into categories and made comparisions among the CBPR members, the CAs and the members of CAG & SWG [21]. Additionally, we made comparisons between the three data sources as a way to triangulate the findings. We presented these findings to the CBPR members and the CAs for their feedback and input prior to finalizing the results.

### Approvals

This study was approved by the Oxford Tropical Research Ethics Committee at the University of Oxford (556 − 20), by the Imperial College Ethics Committee (20IC6420) and by the management of the three existing community-based organizations (CBO) under which the CBPR groups were formed. All participants provided separate written consent to participate in the evaluation component.

## Results

The evaluation phase took place from August to November 2022, with the final stakeholder dissemination meeting in March 2023. The first evaluation meeting was held in August 2022 with participation from six CAs from the three CBPR groups. The second evaluation meeting was held in the afternoon of the same day and included 16 CBPR members from all three groups, without the attendance of CAs. We also conducted in-depth evaluation interviews with seven CBPR members, two CAs and one member of the SWG (*n* = 10) from October to November 2022. In March 2023, a dissemination meeting was organized in Ho Chi Minh city and 70 participants, including the representatives of each CBPR group, SWG, CAG, CAs, researchers from other organizations (community-based, national and international), as well as govenrment officials from the Ministry of Health, the Department of Health of HCMC, and other health-related policy makers attended the full day meeting. The photo exhibit included 32 photos from group members, displayed around the meeting venue, with descriptions written by the group members.

We present the results in four categories starting with participatory impacts followed by collaborative impacts. Then we describe the benefits and challenges outlined by the participants. Finally, we describe the key suggestions that the CAs and members provided for future CBPR research (Table 1). In this manuscript, we present the results related to the CBPR experiences and process; results related to HCV will be presented in a forthcoming manuscript.

**Table 1:** Summary of results for CBPR project evaluation

Category	Description
Participatory impacts: Leadership, collaboration and research growth	Varying levels of engagement and research skills created challenges in the beginning of the project for CAs and members. Practice in research methods and forms of engagement was essential. Over time, the sessions incited excitement.
Collaborative impacts: CBPR as an extended journey together	The CBPR group expanded beyond the individual group to include all CBPR groups in the study. The collaboration provided a bigger picture of their communities and they envisioned a shared agenda.
Benefits: Importance of trust and building on existing community relationships	There are many advantages to working within your own communities including pre-existing trust. Core members of communities understand well the issues that are prevalent in the community. Trust extended into the wider communities.
Challenges: Blurred boundaries between CBPR study objectives and clinical trial aims	There was confusion how the CBPR study linked with the OUCRU clinical trial causing a mismatch between trial requirements and community expectations. However, all participants agreed that a benefit of the study was the linkage to care and treatment for HCV.
Suggestions for future CBPR studies	The OUCRU team should communicate the goals of our organization and the goals of the individual projects more clearly to build and enhance trust among all participants. The CBPR meetings should be conducted more frequently and on an established timeline to encourage participation. The CBPR groups thought it could be beneficial to create similar studies with other populations in the region.

### Participatory impacts over time: Leadership, collaboration, and research growth

Everyone involved in the project had different levels and types of engagement, including operating and facilitating group meetings (CAs), contributing ideas to the discussion topics (most members), supporting other members (CAs and often members), collaborating with partners from other organizations (CAs mostly), and utilizing CBPR tools (most members). At the start of the project, both CAs and CBPR members discussed the challenges of working within their groups due to the novelty of the research approach and their varied experiences with leading or participating in such a group. For example, the CAs needed to explain the study aims and the principles of the CBPR approach to members and, specifically for the PWID and LFR groups, there were very few people who had experiences of participating in a study and no one had ever heard of CBPR prior to this project. For these two groups, understanding the study objectives as well as applying CBPR was a struggle and took time. On the other hand, the MSMTG group had a few members who had some research skills and experience so it was less of a challenge but the principles of CBPR were still difficult to relay.

*It was quite hard in general… I mean, it was hard to understand at first what are the differences between CBPR and ‘normal’ research. I mean, for all research, we always work in groups, now we also are working in groups… what I found most interesting about CBPR is however the tools. I feel we became more active in doing those activities. Normally we tend to listen. Now we can also draw.*

A member of MSMTG group

The CAs of all three groups discussed that the main difficulty in the early phase of the forming the group was figuring out how to facilitate and encourage members to interact and collaborate with each other despite their different backgrounds, priorities, and needs. While the three groups had a shared and very broad priority of identifying and overcoming the challenges of accessing HCV care and treatment, challenges arose when the groups had to narrow down to identify specific issues for their communities. This was one of the first exercises for the groups (i.e. problem identification). The CAs reported that it was difficult because members were using new methods (i.e. based on CBPR principles) to brainstorm and discuss. It took time for the groups to agree regarding the specific problem upon which to focus.

*“I guess it was because people had different problems. I know we are assigned into the same group… like we are in LFR group, everybody knows we are very poor. All of us don’t have stable income. Most are unemployed but that doesn’t mean we have the same issues when it comes to hepatitis C treatment. For some the problem is they don’t have information [about hepatitis]; for others it’s the documents to buy insurance [national health insurance] so they cannot go to hospital.”*

CA of LFR group

Additionally, the first time the group members collected data from their wider community, it was equally challenging because most members had little to no experience of collecting data of any kind. Members reported that they did not feel confident during the initial data collection, however, it became easier due to the support from the CAs and their natural improvisation when they became more acquainted to interview and group discussion techniques. Before collecting data in the wider community, all groups had prepared a set of research questions and tools and practiced with each other. All members were encouraged to identify the research focus/questions and review the tools that they found most relevant and feasible to use in their communities.

*My first time doing an interview was really tough. Even thought Ms M. [CA of LFR group] came with me and sat next to me when I interviewed, I didn’t know how to keep the conversation going. Then Ms. M helped me out. She asked questions with me. The second time I felt more confident, I started with asking about his [the community person] life, and job and wife then the conversation kept go on and on… not too bad I think.*

Member of LFR group

Both CAs and group members revealed that practice was essential and needed to be done through cycles of learning and unlearning, doing and undoing, to the extent that they could grasp understanding about each other and the priority of their groups, including their own priorities.

*The first [thing that] came to my mind about what I learnt is the knowledge about hep C. To be honest I was not sure about the difference between hep B and C. Actually I didn’t care, so I didn’t seek information about it. Now that I know about it, I started to care. I mean, when just one person is aware, the knowledge will spread.*

Member of LFR group

Over time, the CBPR sessions incited excitement and inspired members to form new ideas for subsequent cycles of research. Being a part of the CBPR group, both members and CAs reported that they learnt new skills and became more independent. This was especially true for the PWID and LFR groups since some members had never participated in any kind of research before. They could not imagine that they could have autonomy throughout the whole research process, from identifying research questions to conducting research and suggesting solutions. In addition to research skills, the CBPR members gained more confidence in expressing their opinions, as well as becoming better active listeners to others’ experiences. They reported high levels of satisfaction as they considered themselves to be *helpful* since they were able to reach out to many people in their communities and communicate information about HCV.

*“It was hard and uncommon. It’s uncommon so it’s hard, I mean… I noticed other people did not say that but I will say it: I can talk more during these [CBPR] sessions. Doesn’t mean we cannot give ideas in previous research studies but the environement here is different. People get more comfortable. I and Ms. N [other member] drafted the questions and brought it to community [when they conducted research in their wider community].*

Member of PWID group

Finally, members made several positive comments during the reflection time in the dissemination meeting. They were amazed that a room full of leaders and policy makers would join for a full-day conference to hear about their research results and suggestions for improving access to care and treatment for viral hepatitis.

*I couldn’t sleep at night. I had to prepare for my task, you know… come up to the stage and talk about our groups. I had to practice a lot. You can ask my husband, he can tell you. But today was more than I expected. I am glad I can make it [presenting groups’ results]. I am glad we have them [policy makers, healthcare experts, leaders] coming today. It’s so great that everybody can see each other in person and talk. I am still shaking [laugh] but I am happy that we are doing this.*

Member of PWID group

In addition, other stakeholders also reported that it was meaningful to have community members join a research dissemination meeting. One senior government stakeholder expressed:

“*This is the first time I have been to a health meeting and heard directly from the community*,* the people affected. This method should be applied to HIV and other diseases.”*

### Collaborative impacts: CBPR as an extended journey together

As mentioned, CBPR members were enthusiastic about the characteristics of CBPR methods and this led to collaborative impacts. Members discussed the cyclical nature of CBPR as a “step by step…journey with companions.” Put differently, they emphasized the fact that everyone, from the CAs to the members and also the OUCRU team, worked and supported each other from the very beginning until the last cycle of the project. CBPR members described their participation as “not alone”, and they were ready to try their best to understand the study goals as well as ways to collaborate with each other (i.e. between and among groups).

Among the CAs specifically, participants mentioned that the CBPR study also provided a chance to learn more about other underserved communities’ struggles and needs. For example, the needs from the two other groups that were part of the study, which gave them more understanding and an array of information for future research and cooperation between communities. They also learned how to work with different communities using distinct approaches. For example, the CAs often met with each other to discuss their approaches and how their groups were working through the CBPR process. Applying CBPR, for both members and CAs, allowed them to reconsider their communities’ conditions and resources from a different point of view. They were “in” but also “out”. They were the members of their communities, but then, they also became the “observer” of who and what was in.

*I can listen to what other people think of this disease and the struggles we are facing. I thought we all have the same struggles but then I realized everybody views it differently. I thought I understood my community and the people within it but well… it’s too big. Different people come to this meeting and bring with them different points of view. Now that changed my point of view also. I look at my community and myself differently. I can help myself and help people.*

A member of PWID group

The widened point of view gave them the advantage of seeing a bigger picture of their communities in order to propose more realistic solutions that matched the context. The problems went beyond the individual and they started to think about shared agendas.

### Importance of trust and building on existing community relationships

The evaluation data also provided insightful perceptions from participants about the advantages of forming CBPR groups and practicing CBPR in their own communities. In some instances, the CBPR members from one group knew each other and the CAs before joining the group, therefore there was some pre-existing trust between the CAs and members. This was not always the case because there were members who were introduced into the group by another member. The CAs and even some active members were the gatekeepers of these communities: they were aware of the shared struggles and needs in the community, as well as knowing who might be available to join and collaborate in the study. The trust they established with these people also enabled them to approach and explain the study goal before the first meeting, resulting in their confidence to attend the meetings. Members relayed examples of being taken advantage of in the past under the name of research, but because of the CBPR approach, community members were invited by CAs rather than external researchers and this helped build more confidence in the study.

Additionally, as core members of their communities who understood the context of the issues, the CAs could directly relate to the struggles that members spoke of in meetings. This was particularly helpful during the CBPR activities in which the CAs were in charge of encouraging and sustaining the discussion, confirming with members what they thought were the key take-away points. They always summarized those points at the end of each session to make sure they understood correctly. Besides being the gatekeepers, the CAs also helped to establish and maintain connection between members.

Trust outside of the groups was also important. Some CBPR members, even though they were not CAs for the project, often held facilitation roles in their communities (i.e. consulting with people about transmission of HIV, HCV, and/or harm-reduction services). While it was mentioned that most members found it difficult to partake in this study because they did not have any data collection experience, those members who had previous experience of talking and listening to the community, admitted that this was beneficial during data collection.

*“I guess it was due to my experience of talking with them [community people] about HIV prevention, about syringes and stuff, you know, that in this project, I can go and talk. I don’t know for sure if it’s a way of doing research… the act of talking to people and listening to what they have to say… but when I think of research this way, I feel more comfortable and confident. If collecting stories is the same of collecting research data, then I am doing it.”*

A member of PWID group

The connection and trust, therefore, extended beyond the groups into the wider communities.

### Blurred boundaries between CBPR study objectives and clinical trial aims

The CBPR project was conducted alongside an OUCRU clinical trial, which caused some confusion with both CAs and CBPR members, in particular regarding the CBPR study’s overarching goals. The OUCRU CBPR project team attempted to make it clear that the CBPR project and the clinical trial were separate, and distinct studies, however, the OUCRU trial team recruited for the trial from the CBPR groups. CBPR members as well as the CAs shared that they felt unclear about the CBPR research goals and some even thought that the most important objective was to help the trial recruit participants. The confusion about the conflicting study goals, surprisingly, was only uncovered later in the CBPR project when the members went to apply CBPR within their wider communities. The CBPR members introduced the trial to community members and hinted about the chance of getting cured from HCV, if they joined the trial. This was not necessarily ‘wrong’ because anyone could join the trial if they were eligible and the studies were linked on some levels. However, as some CBPR members misunderstood that recruiting participants for trial was the ultimate goal of this study, then they solely focused on introducing the trial during the CBPR data collection.

*[In the] first couple of meetings, they [members] didn’t realize it was about collecting data but thought it [joining this study] would get them to treatment [at trial study]. Later on they started to understand it’s both. I think it’s good that we are able to do both – now they can both [participant in the] study and be referred to the trial.*

CA of PWID group

Further, the CAs of the LFR group mentioned the number of people they were able to refer to the trial during the evaluation meeting when asked about the study results. An unintended consequence of this was that the CBPR members did not fully explain the trial’s selection criteria and many people went to the hospital to enroll but ended up being excluded from the trial. There was a mismatch between the information provided, trial requirements, and community expectations.

While there was some confusion regarding the aims, all participants who participated in the evaluation component agreed that one of the most practical benefits of joining the CBPR study was the linkage to care and treatment via the trial. While this was not the ultimate goal of the CBPR study, being cured from HCV was indeed an important aspect for the members. Beyond the potential to join the trial, most members reported that they gained more awareness about HCV through educational sessions led by study doctors in the communities.

### Members’ suggestions for future CBPR studies

We recieved multiple suggestions to improve the operational aspects of CBPR projects in the future from participants. First, the OUCRU team should communicate the goals of our organization and the goals of the individual projects more clearly to build and enhance trust among all participants. Second, participants suggested that for future projects, the CBPR meetings should be conducted more frequently and on an established timeline. Part of the reason behind these suggestions was that many of the CBPR meetings were not arranged regularly with a fixed schedule but instead were based on the availability of members and CAs, as well as the local COVID-19 situation. Some participants reflected that it was difficult for them to remember the scope of work as well as the data they collected from community members when there were large gaps in between meetings. Third, CBPR members and CAs desired more trainings about research skills, mental health consultation skills, and information sessions about HCV so that they could not only collect more in-depth data, but also communicate and answer inquiries from the community. The last suggestion was to create similar studies in other geographical and demographical areas, such as with the MSMTG commercial sex workers, middle-age sex workers, and those who have limited financial resources that lost their identity documents and were unable to obtain new ones.

## Discussion

Overall, in the evaluation component of the CBPR project, members described how they and their groups changed over time in leadership skills, in collaborating with other people and groups, and importantly how their research skills improved. They also described their experiences between the three groups, including the other CBPR groups and the academic researchers. These are important impacts from their point of view.

While reflecting on their own journeys with the process, it was apparent that time and commitment were essential for group success. Many of the group members had little to no experience with research and/or leadership and needed time to grow into these roles. Even for those with some experience with research, they were often more familiar with being a participant in research, not leading a study or collecting data. Time was a key feature, as noted elsewhere, including time to understand the project, time to become comfortable with the methods, and time to develop as a group within the community [22]. In addition, the findings about trust and leaning on existing community relationships was highlighted as a necessity for the study’s success. Similar to results from a realist review, trust played varying and dynamic roles in the study: it was a preexisting resource that was used by the groups to engage with communities during the research phase, it was a response among the various partners to the process, and it was also built up over time within and between groups as an outcome of the study [23, 24].

If we just define ‘success’ as assessing whether or not people are better off because of their participation in the research as some CBPR researchers do, (e.g. McIntyre 2014), we can see instances where members relayed positive ways that their groups benefited and adjusted throughout the study period. A clear example of adjustment was during tight COVID-19 restrictions in HCMC. The CAs met together and with the OUCRU researchers to discuss how to shift the aims to address immediate issues that members faced, e.g. helping methadone users access methadone when lockdown was strictly enforced, delivering HIV medications to those who had ran out, and providing food and basic necessities within the community (forthcoming). At this stage, the access to HCV care and treatment was not an immediate concern therefore we adapted the process and made decisions together about the project. This example also demonstrates the authenticity of the relationships between all partners, which is widely discussed as an important component of CBPR [25–28]. Further, two indicators of authentic relationships in CBPR have been documented as being adaptability and shared values [28]. The personal and group growth narratives and self-reflections also evidenced that there were shared values among the partners.

One of the main challenges members spoke about was the confusion between the CBPR study and the clinical trial. Because participatory approaches aim to blur boundaries by its nature, it is not surprising that it caused some confusion. It may have been better to keep the trial separate because the initial goals of the CBPR study were not to increase trial recruitment. But perhaps this linkage provided another route to HCV cure and should be viewed as another component alongside the CBPR study. Studies have been conducted where researchers directly used CBPR as a recruitment tool to reach underserved groups for biomedical research. For example, researchers used CBPR to recruit diverse participants for cancer screening and prevention trials in the United States and it yielded good results [29]. Based on our unpublished trial data, there were many community members who were recruited into the trials, yet many were also excluded, which caused more confusion. The challenge of blurred boundaries was part of the process, and some people were able to access treatment and get cured from HCV. Had these boundaries not been blurred, perhaps the outcome would have been different. Other PAR scholars also reflected that the challenges of PAR are required as part of the ways in which communities change and provide avenues for new ways to understand experiences [17]. Further, if members perceived part of the aim was indeed trial recruitment, then we, as the academic research partners, need to manage the conflicting aims and should not say what should or should not be considered the aim.

### Limitations

There are two main limitations to the evaluation component of the CBPR study. First, the OUCRU team was the primary data collection team for the evaluation component, therefore the experiences and opinions of the OUCRU research assistants and investigators from the academic partner were not explored beyond reflections in the research assistants’ fieldnotes. In future studies, it may be worthwhile to have a CBPR member interview the academic partners on similar topics and integrate it into the analysis. Another limitation is that the evaluation workshops and interviews took place several months before the stakeholder dissemination meeting, therefore we did not collect data on participants’ perceptions except in the fieldnotes from the meeting, noting that the presence of policy makers and government officials may have impacted the participants’ responses. In future studies, it may be better to have the evaluation data collection after the dissemination meeting as this meeting was a key outcome of the project.

## Conclusions

In summary, the CBPR partners from the study described the main impacts over time. These included personal and group improvements in leadership, collaboration, and research skills. The relationships built and maintained led to community members linking with leaders from the community and institutions to share their lived experiences of care seeking and treatment for HCV. In conclusion, the evaluation approach that we co-developed led to both a research reflection on the ‘success’ of the project and enabled participants themselves to reflect on the outcomes and benefits of the study from their point of view.

### Afterward

After the official completion of this project, the CBPR group members of all three groups, along with the CAs and with support from the academic researchers and the stakeholder groups, co-wrote a small seed award grant and obtained funding to continue the project and focus on additional issues related to care seeking within their communities. The three groups are now conducting rounds of research on issues related to accessing health insurance, a cross-cutting theme that was discussed across groups during the main project.

### Electronic supplementary material

Below is the link to the electronic supplementary material.

Supplementary Material 1

## Data Availability

The datasets used and/or analysed during the current study are available from the corresponding author on reasonable request.

## Abbreviations

CA: Community activators
CBPR: Community-based participatory research
CBO: Community-based organizations
CPAR: Critical participatory action research
HCMC: Ho Chi Minh City
HCV: Hepatitis C virus
LFR: Limited financial resources
MSMTG: Men who have sex with men and transgendered individuals
OUCRU: Oxford University Clinical Research Unit
PAR: Participatory action research
PWID: People who inject drugs
SWG: Stakeholder working group
WHO: World Health Organization
