# Supplementary material for: Impact of a community-based participatory research project with underserved communities at risk for hepatitis C virus in Ho Chi Minh City, Vietnam: an evaluation study
Source: Res Involv Engagem. 2024 Aug 7;10:82. doi: 10.1186/s40900-024-00619-6 (PMC11304891; doi:10.1186/s40900-024-00619-6)
Supplement: Supplementary file 1 — Supplementary Material 1 [file 40900_2024_619_MOESM1_ESM.docx]

**Appendix 1: Results of GRIPP2-SF**

| **Section and Topic** | **Item** | **Description** |
| --- | --- | --- |
| **1. Aims** | \|  \| **Report the aim of PPI in the study** \| \| --- \| --- \| | **Pg 2** The aims of the evaluation were to explore the main benefits and challenges in implementing and participating in a CBPR study with underserved communities in Ho Chi Minh City (HCMC), Vietnam and to identify and describe various forms of impact. |
| **2. Methods** | \| **Provide a clear description of methods used for PPI in the study** \| \| \| --- \| --- \| \|  \| | **Pg 3-4** See Methods section.  Major sections include: *Evaluation meetings, In-depth interviews, Reflection at dissemination meeting, Analysis, and Approvals* |
| **3. Study Results** | **Outcomes: Report the results of PPI in the study, including both positive and negative outcomes** | **Pg 5-9** See Results section.  Major sections include: *Participatory impacts over time: Leadership, collaboration, and research growth, Collaborative impacts: CBPR as an extended journey together, Importance of trust and building on existing community relationships, Blurred boundaries between CBPR study objectives and clinical trial aims, Members’ suggestions for future CBPR studies* |
| **4. Discussion and conclusions** | **Outcomes: Comment on the extent to which PPI influenced the study overall. Describe positive and negative effects** | **Pg. 9-11** See discussion section. |
| **5. Critical perspective** | **Comment critically on the PPI in the study, reflecting on the things that went well and those that did not, so others can learn from this experience** | See results sections (from participants’ data): *Blurred boundaries between CBPR study objectives and clinical trial aims, Members’ suggestions for future CBPR studies*  See discussion section, especially limitations section. |
